# Age-Related Clinical Presentation of MOG-IgG Seropositivity in Israel

**DOI:** 10.3389/fneur.2020.612304

**Published:** 2021-01-21

**Authors:** Livnat Brill, Esther Ganelin-Cohen, Ron Dabby, Shira Rabinowicz, Efrat Zohar-Dayan, Netaniel Rein, Eyal Aloni, Yuval Karmon, Adi Vaknin-Dembinsky

**Affiliations:** ^1^Department of Neurology and Laboratory of Neuroimmunology, The Agnes Ginges Center for Neurogenetics, Hadassah Medical Center, Faculty of Medicine, Hebrew University of Jerusalem, Jerusalem, Israel; ^2^Schneider Children's Medical Center, Institute of Pediatric Neurology, Affiliated With Sackler School of Medicine, Tel-Aviv University, Tel-Aviv, Israel; ^3^Department of Neurology, Edith Wolfson Medical Center, Holon, Affiliated With Sackler Faculty of Medicine, Tel Aviv University, Tel-Aviv, Israel; ^4^Pediatric Neurology Unit, Sheba Medical Center, Ramat Gan, Israel. Sackler School of Medicine, The Edmond and Lilly Safra Children's Hospital, Tel-Aviv University, Tel-Aviv, Israel; ^5^Department of Ophthalmology, Barzilai University Medical Center, Ashkelon, Israel; ^6^Department of Neurology, Meir General Hospital, Kfar Saba, Israel

**Keywords:** MOG-IgG, optic neuritis (ON), NMOSD, ADEM, encephalitis, MS, MOG antibody disease (MOGAD), demyelinating diseases

## Abstract

**Introduction:** Myelin oligodendrocyte glycoprotein (MOG) antibody associated disorders (MOGAD) have been recognized over the past 10 years as distinct inflammatory, demyelinating diseases of the central nervous system (CNS). Antibodies against MOG are found mostly in patients with optic neuritis (ON), acute disseminated encephalomyelitis (ADEM), and aquaporin-4 antibody (AQP4-abs)-seronegative neuromyelitis optica spectrum disorders (NMOSD). However, data on the disease course and disability outcomes of these patients are scarce.

**Aim:** To describe clinical and paraclinical features associated with MOG antibodies (abs) in a cohort of patients in Israel, and to assess baseline prognostic features of MOG-ab-associated diseases after a first acute demyelinating event.

**Methods:** MOG-abs were identified in serum using a cell-based assay, and clinical data were collected from the patients' medical records.

**Results:** Of 683 patients with demyelinating diseases tested for MOG-abs, 53 were positive (7.7%), with ON the most common presenting phenotype (68%). The age range of MOG-abs seropositive patients was 1–66 years, with increased prevalence in children (19% compared to 6.7% in adults) (*p* < 0.01). The highest prevalence of seropositivity was observed in children aged younger than 10 years (25.5%), followed by those aged 31–40 years (16.6%).

**Conclusions:** MOGAD are distinct autoimmune diseases that occurs at all stages of life with a significantly higher prevalence in children; the main clinical presenting phenotype in the entire cohort is ON and young children most often presented with ON or ADEM. Our data highlight the need for repeated evaluation of MOG-abs in patients with acquired CNS demyelinating disorders, especially in children under 10 and adults between 31 and 40 years of age.

## Introduction

Myelin oligodendrocyte glycoprotein (MOG) antibody associated disorders (MOGAD) have been recognized in the past 10 years as distinct inflammatory demyelinating diseases of the central nervous system (CNS), characterized by the presence of immunoglobulin G (IgG) class 1 antibodies targeting MOG (1–3). MOG is a component of the myelin sheath uniquely expressed in oligodendrocytes in the CNS, which has been described as a potential target of demyelinating diseases (4, 5). MOG peptides are known to elicit a demyelinating immune response in experimental models of inflammatory demyelinating diseases (6, 7). MOG-abs have been mentioned in the literature for almost 30 years, although their role in demyelinating diseases has not been fully elucidated until this decade (8, 9).

MOGAD are characterized by monophasic or relapsing optic neuritis (ON), myelitis, brainstem, and cerebral cortical encephalitis (1, 10, 11), and in children by monophasic acute disseminated encephalomyelitis (ADEM) and ADEM followed by recurrent ON, multiphasic disseminated encephalomyelitis or AQP4-ab-negative neuromyelitis optica spectrum disorders (NMOSD) (12, 13). The most common phenotype is ON, which is frequently bilateral, as well as ADEM in young children (14, 15). Clinically and radiologically, MOGAD resembles ADEM and NMOSD (2, 10, 16, 17), although MOG-abs does not induce astrocyte injury like AQP4-abs, and MOGAD are considered milder and less relapsing (18, 19). In addition, prognosis is typically favorable compared to both MS and NMOSD, and standard MS treatments such as beta interferon, natalizumab, and glatiramer acetate (20, 21) may exacerbate disease. Attacks are treated with steroids, and those with suboptimal response may be treated with plasma exchange or intravenous immunoglobulin. Treatment response varies between MOGAD patients, and immunosuppressive treatments including rituximab or mycophenolate mofetil, often result in incomplete disease control (10, 22–24). Initial clinical manifestations of MOGAD can occur at any age, with mean age of onset at 25–30 years (4), younger than NMOSD onset, which typically occurs at age 35–45 years. MS and NMOSD are mostly diagnosed in young adults, with relatively few cases in pediatric and elderly patients (~20% of diagnosed patients) (25, 26). In contrast, MOGAD occurs more often among young children (4), similar to ADEM (27). Age of onset has been suggested as an important factor for determining the clinical course and the resulting disability in inflammatory demyelinating diseases. For example, late-onset cases of MS more often present with spinal cord lesions and are associated with rapid disease progression (28–31). Some studies have shown that late-onset NMOSD is predictive of motor disability but also of relatively good visual function. In contrast, pediatric NMOSD has a higher probability of developing visual disability (32, 33).

In this work, we studied detailed clinical characteristics and prognosis for MOG-abs-positive patients in a multicenter cohort in Israel, with an emphasis on clinical manifestations at different ages.

## Materials and Methods

### Ethics

This research was approved by the Hadassah Medical Organization's Ethics Committee.

### MOG Antibody Testing

Serum samples were tested for MOG-abs using the Euroimmune commercial biochip immunofluorescence fixed cell-based assay (clinical specificity of 98.1%) (34, 35). All MOG antibody-positive patients were negative for AQP4 antibodies.

### Case Selection and Data Collection

Clinical information and samples for this study were collected from eight centers in Israel from January 2017 to January 2020. A total of 683 patients with demyelinating disease were tested for MOG-abs, 53 patients were identified as positive. Epidemiologic data, including demographic, disease presentation, disease course, clinical outcomes, oligoclonal bands (OCB), magnetic resonance imaging (MRI) findings and treatments were obtained from medical records. Data were available for 46 MOG-abs positive patients.

Relapses were defined as new neurologic symptoms lasting at least 24 h, accompanied by new neurologic findings, occurring 30 days or longer following the previous attack. The outcome reached after the first attack and at last follow-up visit was evaluated by the Expanded Disability Status Scale (EDSS) score.

### Statistical Analysis

The Chi-square statistic with Yates correction was performed, using GraphPad Prism 7 (GraphPad Software, San Diego, CA).

## Results

### Clinical Characterization of MOG-abs Seropositivity in Israel

Of 683 patients with inflammatory demyelinating disease that were tested for the presence of MOG-abs, 53 (7.7%) were positive (29 males, 24 females). The median age of onset was 22 years (range 1-66 years): 10 years in children (range, 1–18 years), and 35 years in adults (range, 19–66 years).

The most common presenting phenotype in the total cohort was ON (68%) [46.9% bilateral ON [BON], 37.5% unilateral ON [UON] and 15.6% recurrent ON], followed by myelitis (40.4%) and ADEM (19%). Of 46 patients with a median follow-up period of 30 (range 6–126) months, 56.5% of the patients presented with a monophasic disease course while 43.5% experienced recurrent relapses. Thirty-five percent and 58% of the pediatric and adult patients respectively had relapsing disease (range 2–3 and 2–6 relapses for the pediatric and adult patients, respectively, *p* = 0.14, follow-up data were not available for 13%).

Clinical and demographic data of the patients, according to age at disease onset, are summarized in Table 1.

**Table 1:** Clinical and demographic characterization of MOG-IgG patients.

**Age (years)**		**1–10**	**11–20**	**21–30**	**31–40**	**41–50**	**>50**	**Total cohort**
F/M		6/7	3/9	4/3	7/8	1/2	2/0	23/29
ON		8/12 (67%)	9/11 (82%)	2/5(40%)	9/14 (64%)	2/2 (100%)	2/3 (67%)	32/46 (69.5%)
Myelitis		4/12 (33%)	3/11 (27%)	3/5 (60%)	7/14 (50%)	2/2 (100%)	1/3 (33%)	19/46 (41%)
ON and Myelitis		1/12 (8%)	1/11 (9%)		2/14 (14%)	2/2 (100%)		6/46 (13%)
MRI Brain	Normal	5/12 (42%)	7/11 (64%)	3/5 (60%)	10/14 (71%)	1/2 (50%)		29/46 (63%)
	ADEM	4/12 (33%)	3/11 (27%)	1/5 (20%)	1/14 (7%)		3/3(100%)	8/46(17%)
	NMOSD like (36)	1/12 (8%)		1/5 (20%)	2/14 (14%)			3/46 (6.5%)
	MS like		1/11 (9%)			1/2 (50%)		2/46 (4%)
Disease course	Relapsing	3/12(25%)	5/11 (45%)	1/5 (20%)	9/14 (64%)	2/2(100%)		20/46(43%)
	Monophasic	8/12 (67%)	6/11 (54%)	4/5 (80%)	5/14 (36%)		3/3(100%)	26/46 (56%)
OCB		1/11 (8%)	1/11(9%)	1/5 (20%)	2/14 (14%)		1/3 (33%)	6/46 (13%)
Diagnosis	ON	6/12 (50%)	6/11 (54%)	2/5 (40%)	5/14 (36%)		2/3 (67%)	21/46 (46%)
	ADEM	6/12 (50%)	3/11 (27%)					9/46 (19.5%)
	Myelitis		2/11 (18%)	2/5 (40%)	4/14 (29%)		1/3 (33%)	9/46 (19.5%)
	NMOSD like			1/5 (20%)	3/14 (21%)	1/2 (50%)		5/46 (11%)
	MS like				2/14 (2%)	1/2 (50%)		3/46 (6.5%)
MOG-abs seropositive		13/49(26%)	12/97 (12%)	7/136 (5%)	15/90 (17%)	3/98 (3%)	3/107 (3%)	53/683 (7.8%)

### Age-Dependent Seroprevalence and Clinical Presentations of MOGAD Patients

**Figure 1:**
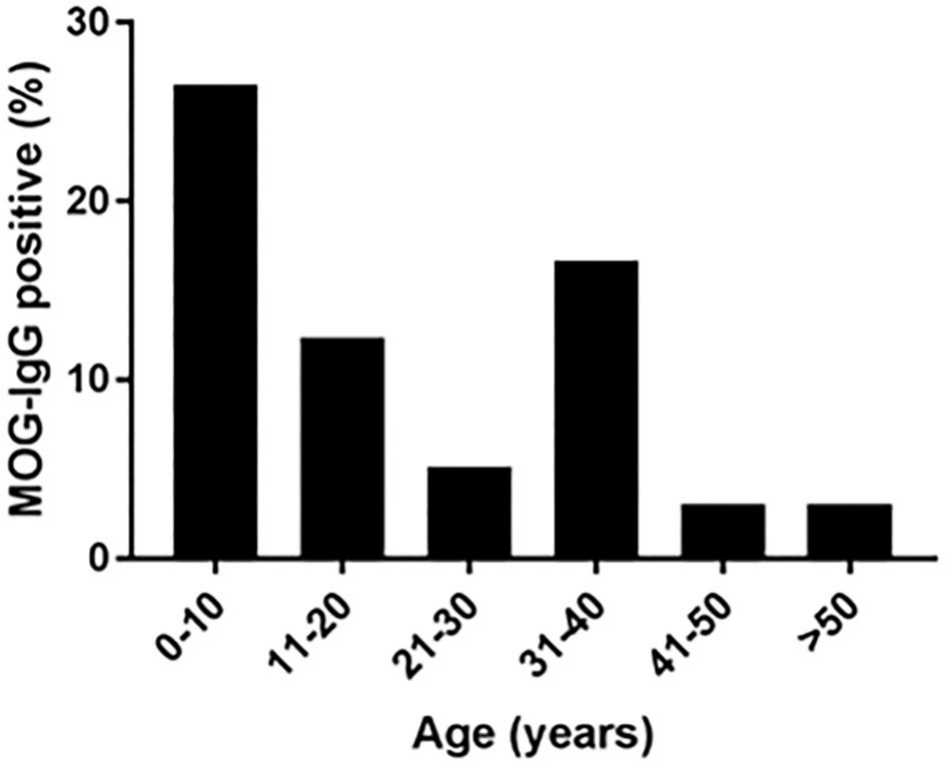
Age at onset in MOG-abs-positive patients in Israel. % of MOG-abs-seropositive patients of those patients with demyelinating disease tested for MOG-abs.

The age of the tested patients ranged from 1 to 82 years. MOG-abs seropositive patients were found between the ages of 1 and 66 years. The highest prevalence of seropositivity was observed in children aged 1–10 years (25.5%), followed by the age group 31–40 years (16.6%), compared to 12% between ages 11–20, 5% at ages 21–30 and 3% above age 41 years (Figure 1).

#### Increased Occurrence of MOG-Abs Seropositivity in Children

**Figure 2:**
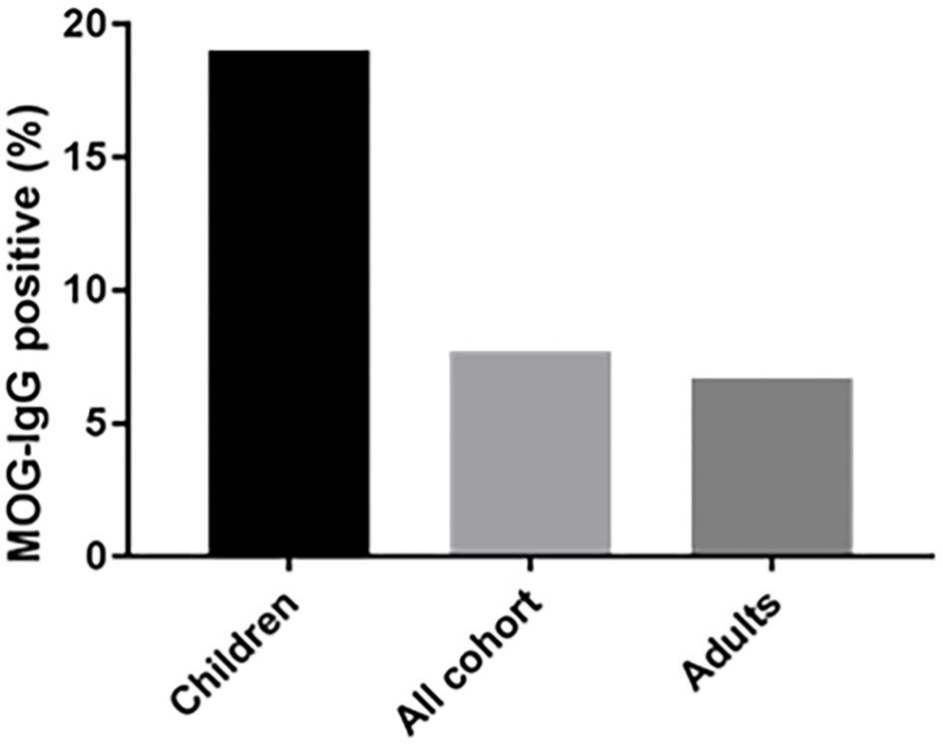
The seroprevalence of MOG-abs is age-dependent. The mean percentages of MOG-abs-positive patients are shown for each subpopulation.

Of the 683 samples that were sent to our laboratory for MOG-abs testing, 121 samples were from children (≤ 18). A total of 23 samples from pediatric patients were positive (19%) compared to 30 samples from 447 adult patients (age > 18) (6.7%) (*P* < 0.01) (Figure 2). 43.4% of the MOG-abs-positive patients were aged 18 and under, compared to 15% in the MOG-abs-negative population. The highest prevalence of MOG-abs seropositivity in children was found between ages 1–10 (25.5%).

**Figure 3:**
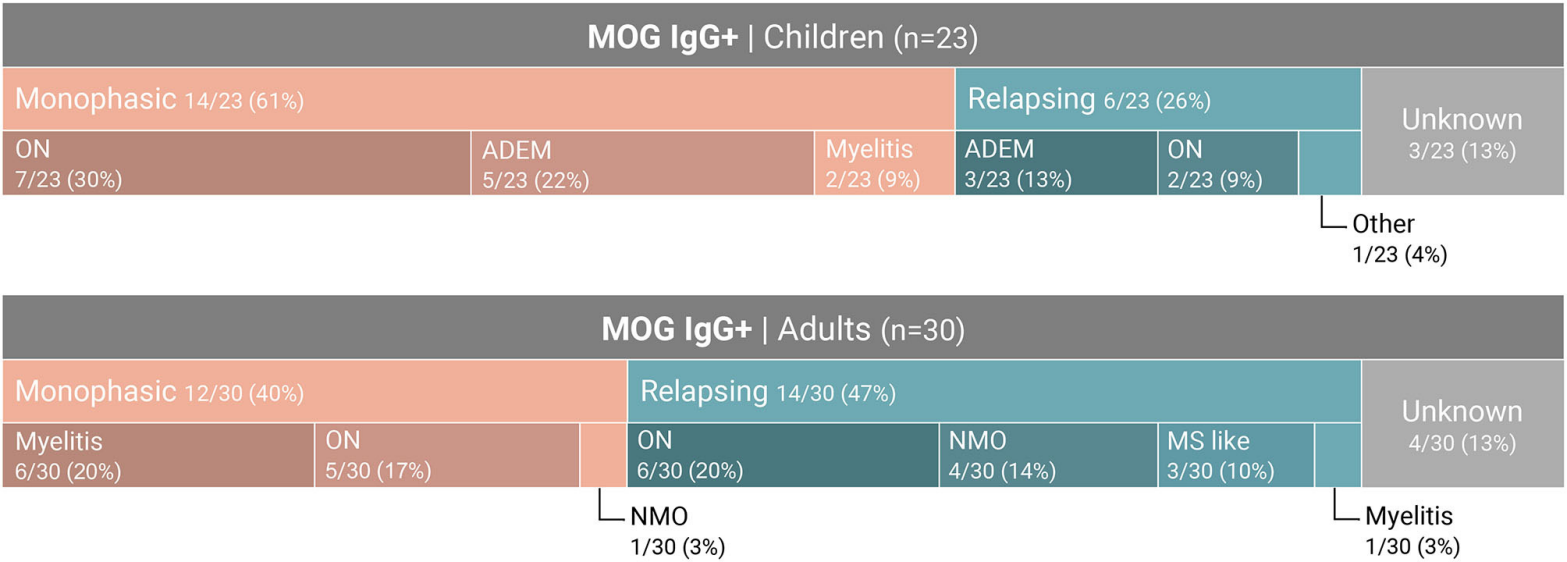
Distribution of seropositive MOG-abs among pediatric and adults clinical phenotype.

On examination of MOGAD presentation, we identified distinct disease characteristics related to the age of onset (Figure 3). ON is the most common clinical presentation in our cohort (68%, *p* < 0.01) (bilateral in almost half), and is seen frequently in both pediatric and adult patients (65% and 66.7% respectively). 61% of the pediatric patients presented with a monophasic disease course, whereas a relapsing course is more common among patients aged over 30 years (58%, *p* = 0.15). No age-dependent differences were seen regarding the presence of OCBs (7%-20%, *p* = 0.8).

Twelve patients presented with myelitis only, 75% of whom followed a monophasic disease course (9/12); seven of these nine patients are aged 25–40 years. Out of 13 adults (age 21–50 years) presenting with relapsing disease, four patients aged 36–42 years (31%) were diagnosed initially as NMOSD-like, presenting with both ON and myelitis. Three more patients (23%) presented with typical MS on brain MRI. One patient presented with encephalitis and status epilepticus.

Thirty-eight percent of MOG-abs seropositive patients under age 18 were diagnosed initially as ADEM (8/21), most of whom had a monophasic disease course (5/8, 62.5%). Patients presenting with ADEM features were younger (median age 8 years).

#### Late Onset MOGAD

One hundred and seven samples in our cohort were obtained from patients above the age of 50 years. Three patients, aged 50, 65, and 66 years, were positive for MOG antibodies. All three presented with a monophasic disease course and normal brain MRI. Two presented with ON and one with myelitis. Clinical follow up was 30 months on average (1.5, 1, and 5 years for patients aged 50, 65, and 66 respectively).

## Discussion

MOGAD are newly identified CNS inflammatory conditions, associated with attacks involving the optic nerve, spinal cord, brainstem and the brain. Currently, there are no accepted diagnostic criteria for this disease, although two recent publications have proposed recommendations for diagnosis (14, 21).

We studied a cohort of 53 MOG-abs seropositive patients from multiple centers in Israel and identified MOGAD at all stages of life, from ages 1–66 years, with a significantly higher prevalence in children and also in young adults in their 30s, and with distinct clinical manifestations varying with age. Within the entire cohort of 683 patients, 121 children (≥18 years) with inflammatory demyelinating disease were tested for MOG-abs and 23 were found positive, for a prevalence of 19% compared with 6.7% in adults and 7.8% in the general population (Figure 3). By decade, the highest prevalence of MOG-abs seropositivity was seen between the ages of 1 and 10 years (25.5%) followed by 31–40 years (16.6%), representing a higher prevalence at younger ages compared to patients positive for AQP4-abs (37).

The average age of disease onset of MS is the early 20s, with decreased prevalence in children and the elderly (29, 38). NMOSD usually presents in the fourth decade; in accordance with MS, it is less frequent in children and elderly (32, 33). However, MOGAD age of onset was found to be much younger, and prevalence is higher among children, similar to that seen in ADEM (27). About 30% of the patients with initial diagnosis of ADEM in our cohort were found to be positive for MOG-abs, and 40% of the children positive for MOG-abs clinically resemble ADEM, second only to ON presentation.

Higher prevalence of MOGAD in children has been reported worldwide. Large studies conducted in Europe detected MOG-abs in 22–30% of children with acquired demyelinating disease. These studies have also demonstrated that MOG-abs are present in children more frequently than AQP4-abs (3–6%) (10, 12, 39–41).

The clinical phenotype associated with MOG-abs seropositivity in our cohort varied with age from ADEM in children (40%) to opticospinal (optic neuritis, myelitis and brainstem encephalitis) in adults. Early onset (<18 years) and late onset (>50 years) MOGAD were characterized by monophasic course (14/20 and 3/3, respectively), while most young adults presented with a recurrent course (64% for patients aged 30–40 years). Rare phenotypes of MOGAD, such as encephalitis and Leukodystrophy-like phenotype, have been described (14, 15). In our cohort, one patient presented with encephalitis and status epilepticus. Unlike in MS we did not see a chronic progressive course in any of the MOG-abs seropositive patients. In accordance with our data, de Mol et al. (40) recently described a distinct distribution of clinical phenotypes of MOGAD between adults and children in a typical western European country. The most common phenotypes in children were monophasic ADEM, followed by ON and NMOSD, whereas in adults, ON was the most common phenotype, followed by myelitis and NMOSD.

A female predominance is common in autoimmune disease. In NMOSD there is a female predominance of 9:1 (42–44). However, this ratio is not seen in children and elderly patients with AQP4-abs (43, 44). In our cohort of MOGAD patients, we detected a sex ratio of 1:1 in the adult population, which is more similar to that found in AQP4-ab-negative NMOSD (1:1.9) (45) and in classical MS (3:1) (46). Patients presenting with myelitis were mostly men (6/7). There were no other sex-related differences in disease presentation or course.

Reliable tests for MOG-abs have only become available in recent years (34, 35). For this reason, most of the patients initially received other diagnoses. 35% of the young adult (20–40 years old) positive patients in our cohort were initially diagnosed with MS or NMOSD-like, while 40% of patients aged 18 years or younger were initially diagnosed as ADEM. Children presenting with ON or ADEM and young adults with monophasic or relapsing course who test negative for AQP4-abs are at high likelihood of MOG-abs seropositivity (1, 47). This may have significant implications for subsequent follow-up and treatment.

Although there are no evidence-based guidelines for the treatment of MOGAD (1, 14), and data regarding the efficacy of immunotherapy is limited, accurate diagnosis is important. There are reports supporting distinction of treatment strategies from those typically used for MS, and NMOSD (20–22). As in MS and NMOSD, MOGAD patients are particularly responsive to steroids, plasma exchange or treatment with IVIG for acute attacks. However, while MS is frequently treated with disease-modifying immunotherapy, case series indicate that several MS treatments (interferon beta, glatiramer acetate and natalizumab) do not suppress relapses and might exacerbate MOGAD (20, 21, 23, 24, 48). B cell-depleting therapies that are known to be effective in MS (ocrelizumab, rituximab) (49) and NMOSD (rituximab) (50) are recommended as a relapse preventive therapy in MOGAD. However, several groups recently reported the occurrence of relapses during rituximab treatment. These relapses were attributed to re-occurrence of B cells (21).

Findings of the present study must be considered within the framework of the study's limitations. Limitations include, the relatively small cohort size and short follow-up period. Increasing the sample size and extending the duration of follow-up is important and will confirm and strengthen our results.

In conclusion, we observed a high prevalence of MOG-abs positivity in young children who presented with ON and/or ADEM. As in AQP4-abs, we also observed patients with MOG-abs in older adults, mainly presenting with a monophasic disease course. Testing patients presenting with various types of acquired demyelinating syndromes for MOG-abs may assist with clinical decision-making.

## Data Availability Statement

The original contributions presented in the study are included in the article, further inquiries can be directed to the corresponding author.

## Ethics Statement

The study was reviewed and approved by The Hadassah Medical Organization's Ethics Committee. Written informed consent to participate in this study was provided by the participants.

## Author Contributions

LB preformed the tests, analyzed the data, and wrote the manuscript. EG-C contributed samples and clinical data for the study and to the final version of the manuscript. RD, EZ-D, EA, NR, and YK contributed samples and clinical data for the study. SR contributed samples and clinical data and to final version of the study. AV-D contributed samples and clinical data, contributed to data analysis, and manuscript writing. All authors contributed to the article and approved the submitted version.

## Conflict of Interest

The authors declare that the research was conducted in the absence of any commercial or financial relationships that could be construed as a potential conflict of interest.
